# Cost-effectiveness analysis of tuberculosis screening in diabetic patients in China: a decision-analytic Markov model

**DOI:** 10.3389/fpubh.2025.1696952

**Published:** 2026-01-05

**Authors:** Hui-Wen Song, Yong Lin, Tu-Er Wan, Chen-Fan Zhang, Jin-Shui Pan

**Affiliations:** 1 Department of Hepatology, First Affiliated Hospital of Fujian Medical University, Fuzhou, Fujian, China; 2 Department of Infectious Diseases, Sanming First Hospital Affiliated to Fujian Medical University, Sanming, Fujian, China; 3 Department of Hepatology, National Regional Medical Center, Binhai Campus of the First Affiliated Hospital, Fujian Medical University, Fuzhou, Fujian, China; 4 Department of Radiology, Sanming First Hospital Affiliated to Fujian Medical University, Sanming, Fujian, China

**Keywords:** cost-effectiveness, tuberculosis, diabetes, latent tuberculosis screening, Markov model

## Abstract

**Objective:**

To establish a pharmacoeconomic model to evaluate the cost-effectiveness of various screening strategies for latent tuberculosis infection (LTBI) in older diabetic patients and provide evidence for health policy formulation.

**Methods:**

A decision tree-Markov model was constructed to simulate the LTBI screening process for 10,000 diabetic patients aged 60 years and older, analyzing the costs and health utilities of different screening strategies.

**Results:**

The incremental cost-effectiveness ratio (ICER) of the traditional tuberculin skin test (TST) strategy was significantly lower than the willingness-to-pay threshold, indicating its economic advantage. Meanwhile, the economic benefit of the new recombinant tuberculosis fusion protein skin test (C-TST) compared to TST was not significant, and the interferon-gamma release assay (IGRA) was considered the least cost-effective option due to its high cost. One-way sensitivity analysis identified key parameters that affect the economic viability of screening strategies, such as non-tuberculous mortality rates by age group, LTBI mortality rates, and annual medical costs associated with diabetes. When the non-tuberculous mortality rate reached a certain threshold, the economic viability of all screening strategies was impacted. Additionally, probabilistic sensitivity analysis indicated that TST had a high probability (70%) of being the most cost-effective screening option at a common willingness-to-pay threshold.

**Conclusion:**

Screening for LTBI in older diabetic patients is a cost-effective approach. The strategy should take into account economic conditions and healthcare resource allocation in various regions to enhance the effectiveness of public health interventions.

## 1 Introduction

The prevalence of diabetes mellitus (DM) in China has been steadily increasing, rising from 10.9% in 2013 to 12.4% in 2018–2019, with the prevalence in the population aged 60 and over, approaching or exceeding 20% (1). DM patients are at high risk for progression from latent tuberculosis infection (LTBI) to active tuberculosis, with a risk of pulmonary tuberculosis being three times that of non-diabetic patients (2). This risk is particularly pronounced in older diabetic patients, where the combined effects of aging and metabolic disorders substantially increase disease susceptibility and expedite the deterioration of health-related quality of life (3, 4). Older individuals with diabetes frequently experience a diminished quality of life due to complications. Screening and treatment for LTBI can prevent the onset of tuberculosis (TB), thereby extending life expectancy and reducing mortality within this demographic (5). China bears a substantial burden of LTBI, with over one-fifth of diabetic patients also suffering from LTBI (2). On a global scale, China and India have the highest numbers of LTBI cases, each reaching approximately 350 million, followed by Indonesia (approximately 120 million) (6, 7), highlighting the urgency for targeted screening.

Although LTBI screening and treatment are core measures in the WHO’s strategy to end tuberculosis, the economic trade-offs of screening strategies remain controversial (8). The traditional tuberculin skin test (TST) is widely used in resource-limited areas due to its simplicity and low cost, but its lack of specificity may exacerbate misdiagnosis (9); the interferon-gamma release assay (IGRA), while having high specificity and requiring only a single blood draw, is severely limited in grassroot applications due to its high reagent cost (approximately 300–500 yuan per test) (10). The new recombinant tuberculosis fusion protein skin test (C-TST), with significant cost advantages (approximately 1/6 the cost of IGRA, at around 50 yuan per dose) and no laboratory support required, is a potential alternative. However, its consistency with IGRA and the risk of adverse reactions need further validation (11).

To accurately capture the long-term clinical outcomes and associated costs of different screening strategies, a decision-analytic model that reflects the natural history of tuberculosis is essential. The Markov model is particularly suited for this purpose, as it is adept at simulating the progression of chronic diseases over a lifetime horizon (12). This study employs a Markov model comprising distinct health states to project the accumulated quality-adjusted life years (QALYs) and associated costs for each strategy. This approach enables a comparative assessment of cost-effectiveness that takes into account the evolution of disease risk and intervention effects over time.

Existing studies have notable limitations, lacking cost-effectiveness analyses of tuberculosis screening, specifically for the older DM population in China (13, 14). Most models rely on foreign data while neglecting the specifics of China’s payment capacity (e.g., insufficient insurance coverage) and the uneven distribution of healthcare resources (e.g., higher screening costs in rural areas) (15, 16). This study is the first to systematically compare the cost-effectiveness of three LTBI screening strategies (TST, C-TST, and IGRA) in the ≥60-year-old DM population based on grassroots medical cost data from the Fujian Sanming healthcare reform pilot—a frontier area for national medical insurance payment reform (17). It provides decision-making evidence for resource-diverse regions (e.g., urban vs. rural areas) and offers evidence-based support for the national “tuberculosis-diabetes co-management” plan to integrate comorbidity intervention measures (18).

## 2 Methods

### 2.1 Decision tree-Markov model

A decision tree-Markov model was developed in TreeAge Pro 2022 to assess the cost-effectiveness of three LTBI screening strategies in a diabetic cohort aged ≥60 years. The three screening strategies compared were the TST, with purified protein derivative (PPD) as the reagent at a concentration of 1 mL:50 IU (Beijing Xiangrui Biologicals Co., Ltd., Beijing, China); C-TST, which uses the recombinant *Mycobacterium tuberculosis* fusion protein (EC) at a concentration of 0.3 mL per vial (Anhui Zhifei Longcom Biologic Pharmacy Co., Anhui, China); and the IGRA, specifically QuantiFERON®-TB Gold Plus from QIAGEN GmbH, Germany.

A positive result for the C-TST is defined by a diameter of redness or induration measuring ≥5 mm at 48 h post-injection. Similarly, the TST is considered positive with an induration diameter of ≥5 mm at 48 h post-injection. The results of the IGRA are classified as positive, negative, or indeterminate. In the event of an indeterminate result, a repeat IGRA test is conducted; if the second test also yields an indeterminate result, it is treated as positive, necessitating further investigation to determine if active tuberculosis (ATB) is present (19).

**Figure 1:**
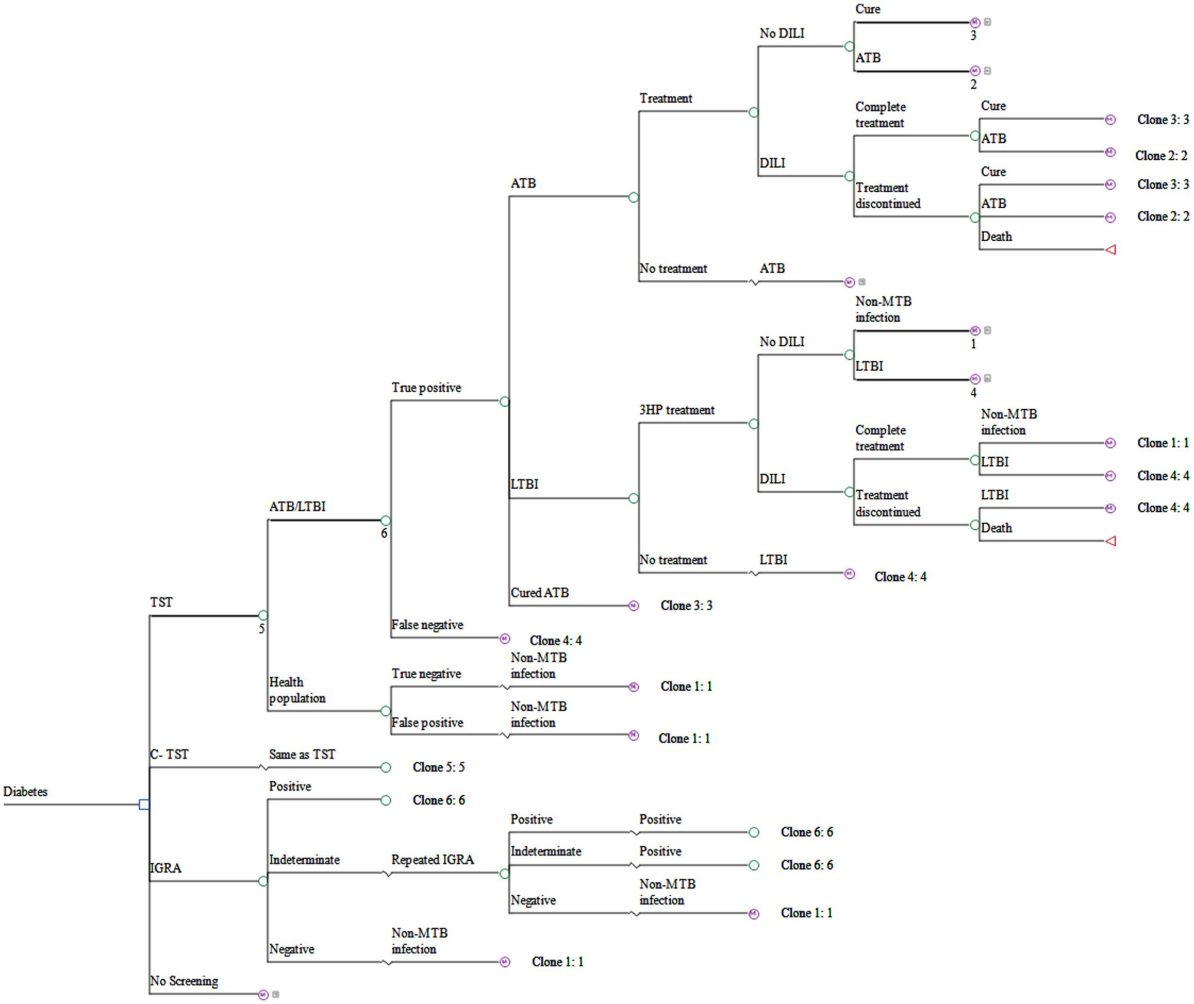
Decision tree-Markov model for screening and treating latent tuberculosis infection in diabetic patients. ATB, active tuberculosis; Cured ATB, cured active tuberculosis; C-TST, creation tuberculin skin test; DILI, Drug-induced liver injury; IGRA, interferon-gamma release assay; LTBI, latent tuberculosis infection; Non-MTB, not infected with *Mycobacterium tuberculosis*; TST, tuberculin skin test; 3HP treatment, isoniazid plus rifapentine administered once weekly for 12 weeks.

The general model structure, shown in Figure 1, along with Supplementary Figures S1–S3, details the standard clinical screening and treatment pathway in China. The decision tree phase simulated the screening process. Patients with a positive test result underwent subsequent confirmatory diagnostics (e.g., imaging, laboratory tests) to distinguish between ATB and LTBI (Supplementary Figure S1). Those diagnosed with ATB received anti-TB treatment (Supplementary Figure S2), while those with LTBI received preventive therapy (Supplementary Figure S3). Patients testing negative received no intervention. Following this initial phase, patients entered a Markov model with annual cycles to simulate long-term outcomes.

### 2.2 Natural history model of tuberculosis

The disease progression was described using five mutually exclusive states: non-infected with *Mycobacterium tuberculosis* (Non-MTB), LTBI, ATB, cured active tuberculosis (Cured ATB), and death (Supplementary Figure S4). The criteria for each state are defined as follows: Non-infected individuals must not exhibit ATB, LTBI, or a history of previous treatment, and must show no evidence of infection across all tests. LTBI is characterized by the exclusion of ATB and cured ATB, with at least one of the three diagnostic tests returning a positive result. ATB diagnosis requires the exclusion of other diseases and adherence to the “WS 288-2017 Tuberculosis Diagnosis Criteria” (20). Cured ATB requires exclusion of ATB and confirmation of a documented history of tuberculosis, along with the completion of anti-tuberculosis treatment. Death is considered an absorbing state in this context.

For the cost-effectiveness analysis of LTBI screening, we simulated an initial cohort of 10,000 diabetic patients aged 60 years. The initial distribution of health states in the queue was determined based on epidemiological parameters, such as the prevalence of LTBI and ATB. Each individual’s health risk—defined by their TB status and treatment—remained constant until a change in status or treatment occurred. In each cycle, individuals occupied one of the following mutually exclusive states: non-MTB, LTBI, ATB, cured ATB, or death. Costs and QALYs were accumulated according to the time spent in each state over the simulation.

### 2.3 Model parameters and assumptions

The model used a 1-year cycle and ran for 19 years, covering the average expected lifespan of the cohort up to the endpoint of 79 years. The screening strategy was set to occur every 2 years, targeting high-risk older diabetic populations (as recommended by the CDC in the U.S.) (21). The study was conducted from the health system perspective, accounting only for direct medical costs, such as screening test costs (TST/IGRA/C-TST), diagnostic follow-up costs (such as complete blood count, biochemical tests, chest X-rays, computed tomography, sputum smear, *Mycobacterium tuberculosis* culture, and nucleic acid detection), preventive treatment costs, and active tuberculosis treatment costs (limited to standard treatment for drug-sensitive pulmonary tuberculosis for 6 months), while excluding indirect costs and costs for drug-resistant tuberculosis (due to significant regional differences in multi-drug resistant tuberculosis without extensive drug resistance (MDR-TB) regimens). Costs and utilities were discounted at an annual rate of 3%, with a half-cycle correction applied.

We assume that all individuals who receive TST/C-TST will return promptly to have the skin test results read. It is assumed that individuals diagnosed with LTBI but not progressing to ATB do not incur health losses during their infection period. Considering the immunodeficiency in older diabetic patients, we assume that LTBI and ATB patients do not have the potential for spontaneous resolution. Patients who test positive in the screening will undergo subsequent examinations to determine whether they are ATB patients through microbiological or clinical judgment. ATB treatment follows the 2HRZE+4HR regimen (2 months of Isoniazid, Rifampin, Pyrazinamide, and Ethambutol, followed by 4 months of Isoniazid and Rifampin) (22). During treatment, patients have monthly follow-up visits, with each visit including assessments of complete blood count, liver function, kidney function, and uric acid levels. Prophylactic treatment follows the 3HP regimen (Isoniazid plus Rifapentine once a week for 12 weeks) (23), with monthly follow-up visits during treatment, each including assessments of complete blood count, liver function, and kidney function. Furthermore, it is assumed that adverse reactions occurring during prophylactic and anti-tuberculosis treatments are solely due to drug-induced liver injury (DILI).

### 2.4 Data sources

Model parameters, health utility values, and other relevant variables were sourced from published literature, as detailed in Table 1. The costs associated with screening and treatment were examined through on-site investigations. Data on medical institution charges were selected from the researcher’s region, which is at the forefront of the national medical insurance payment reform pilot.

**Table 1:** Parameters used in the decision tree-Markov model.

Variables	Range for sensitivity analysis			PSA	Source
	Baseline value	Lower limit	Upper limit		
Screening variables					
TST sensitivity	0.914	0.891	0.933	Beta (626.60, 58.96)	(24)
TST specificity	0.544	0.489	0.597	Beta (176.61, 148.04)	(24)
C-TST sensitivity	0.912	0.89	0.932	Beta (638.39, 61.60)	(24)
C-TST specificity	0.697	0.645	0.745	Beta (225.68, 98.11)	(24)
IGRA sensitivity	0.921	0.899	0.939	Beta (643.17, 55.17)	(24)
IGRA specificity	0.761	0.712	0.805	Beta (245.66, 77.15)	(24)
IGRA uncertain incidence	0.0059	0.0027	0.0102	Beta (9.58, 1614.1)	(41)
Proportions (%)					
Prevalence of ATB	0.5437	0.3423	0.7451	Beta (29.39, 5377.04)	(42)
Prevalence of LTBI	20.34	15.63	25.06	Beta (56.54, 221.43)	(43)
Cured ATB	2.370	1.896	2.844	Beta (95.18, 3920.89)	(42)
Received 3HP treatment	88.4	80.5	96.3	Beta (54.93, 7.21)	(44–47)
Received ATB therapy	76.0	60.8	91.2	Beta (22.26, 7.03)	(48)
DILI associated with 3HP treatment	11.5	1.9	41.5	Beta (1.03, 7.94)	(49)
DILI associated with ATB treatment	9.82	5.40	11.03	Beta (41.84, 384.22)	(50, 51)
Discontinuing 3HP treatment due to DILI	1.7	0.5	4.9	Beta (2.25, 129.97)	(49)
Discontinuing ATB treatment due to DILI	0.86	0.69	1.03	Beta (90.51, 10434.46)	(51)
Death from DILI associated with 3HP treatment	0.7	0.0	5.2	Beta (0.27, 38.62)	(49)
Death from DILI associated with ATB therapy	0.24	0.24	7.14	Beta (0.02, 6.71)	(50, 52)
Transition probabilities (%)					
Incidence of LTBI	0.31	0.21	0.49	Beta (19.55, 6286.37)	(53)
Incidence of ATB	0.2506	0.1278	0.3735	Beta (17.40, 6925.27)	(42)
From LTBI (treated) to Cure	75	60	90	Beta (23.28, 7.76)	(54, 55)
From LTBI (treated) to ATB	0.19	0.07	0.32	Beta (10.01, 5256.74)	(56)
From LTBI (untreated) to ATB	0.49	0.30	0.67	Beta (29.49, 5989.25)	(56)
Mortality (post-ATB treatment)	3.76	3.04	4.60	Beta (85.00, 2175.64)	(57)
Recurrence rate for cured ATB	1.93	0.39	5.60	Beta (2.01, 102.23)	(42)
Cure rate for complete ATB treatment	95.0	57.1	96.6	Beta (3.49, 0.18)	(58–60)
Cure rate for discontinued ATB treatment	40	30	70	Beta (57.27, 85.91)	(61)
ATB (untreated) mortality	12.0	9.6	30.7	Beta (4.26, 31.23)	(62)
Non-MTB mortality (60–64 years)	0.6737	0.5390	0.8085	Beta (99.32, 14642.86)	(63)
Non-MTB mortality (65–79 years)	2.9931	2.3945	3.5917	Beta (96.98, 3143.03)	(52)
LTBI mortality (60–64 years)	0.6737	0.5390	0.8085	Beta (99.32, 14642.86)	(63)
LTBI mortality (65–79 years)	2.9931	2.3945	3.5917	Beta (96.98, 3143.03)	(63)
Utility weight					
LTBI (untreated)	0.97	0.95	1.00	Triangular (0.95, 0.97, 1.00)	(64)
LTBI adopts Prophylactic treatment					
No DILI	0.97	0.95	1.00	Triangular (0.95, 0.97, 1.00)	(64)
DILI	0.94	0.75	1.00	Triangular (0.75, 0.94, 1.00)	(64)
ATB					
ATB (untreated)	0.82	0.65	0.93	Triangular (0.65, 0.82, 0.93)	(64)
ATB (cured)	0.94	0.87	1.00	Triangular (0.87, 0.94, 1.00)	(64)
Costs (CNY)					
TST	8.00	5.25	27.50	Triangular (5.25, 8.00, 27.50)	Field investigation
C-TST	33.53	26.82	40.24	Triangular (26.82, 33.53, 40.24)	
IGRA	293.38	279.00	1090.00	Triangular (279.00, 293.38, 1090.00)	
ATB treatment (6 months)	1949.8	1572.4	2327.2	Triangular (1572.4, 1949.8, 2327.2)	
ATB treatment (interrupted)	958.0	603.2	1312.8	Triangular (603.2, 958.0, 1312.8)	
3HP treatment	631.80	527.56	736.04	Triangular (527.56, 631.80, 736.04)	
3HP treatment (interrupted)	390.00	305.92	474.08	Triangular (305.92, 390.00, 474.08)	
Treatment for drug-induced hepatitis	219.62	124.05	240.50	Triangular (124.05, 219.62, 240.50)	(65)
Annual medical costs for people with diabetes	3726.0	2980.8	4471.2	Triangular (2980.8, 3726.0, 4471.2)	(66)

3HP, Isoniazid + Rifampicin for 3 months; ATB, Active tuberculosis; DILI, Drug-induced hepatitis; LTBI, Latent tuberculosis infection; Non-MTB, Non-tuberculosis infection; QALYs, quality-adjusted life years; TST, tuberculin skin test; C-TST, creation tuberculin skin test.

The diagnostic tests employed in this study, such as C-TST, IGRA, and TST, are based on sensitivity and specificity data obtained from a multicenter, double-blind, randomized controlled clinical trial conducted in China (24). This trial encompassed patients with ATB, suspected pulmonary tuberculosis, and non-tuberculous pulmonary disease. The results showed that the diagnostic efficacy of the C-TST was similar to that of the Diaskin test® and the C-TB skin test, respectively (11, 25). The IGRA exhibited high diagnostic accuracy in this study, with an area under the curve (AUC) of 0.84, corroborating the outcomes of a global meta-analysis (26). Conversely, the TST demonstrated low specificity, measured at 54.4%, which is consistent with previous literature (26). Moreover, our study incorporated the sensitivity and specificity parameters of the IGRA derived from TB-specific enzyme-linked immunospot assay (T-SPOT.TB) results in the model construction. This decision is based on the methodological similarities between T-SPOT.TB and QuantiFERON-TB Gold (QFT), as both assays measure T-cell immune responses triggered by TB-specific antigens. Previous research has demonstrated that the diagnostic efficacy of these two assays is comparable (27). This methodological approach ensures consistency in parameter sources and mitigates the potential heterogeneity that could arise from integrating data obtained through different testing methodologies.

### 2.5 Sensitivity analysis

In one-way sensitivity analysis, if parameters had a 95% confidence interval, the upper and lower limits were taken as the boundaries of that interval; if only point estimates were available, the upper and lower limits were taken as point estimates ±20%, with the upper limit capped at 1 if it exceeded 1 after adjustment. Cost parameters were directly adopted if maximum and minimum values were available; otherwise, the average value ±20% was used. The discount rate was adjusted by ±20% from the baseline.

Probabilistic sensitivity analysis was conducted using a Monte Carlo simulation with 1,000 iterations, where probability parameters followed a Beta distribution and health utility values and cost parameters followed a triangular distribution. Willingness to pay (WTP) refers to the maximum cost that can be accepted for each QALY gained, which can be used to assess the value of various screening programs. The WTP threshold was set at one to three times the per capita gross domestic product (GDP) of Fujian Province in 2024 (28), i.e., from 137,920 yuan to 413,760 yuan. To evaluate the cost-effectiveness of this study using WHO guidelines, the incremental cost-effectiveness ratio (ICER) was compared to the per capita GDP. The following criteria apply: (1) ICER < 0 means the program saves costs and is most cost-effective; (2) 0 < ICER < per capita GDP indicates high cost-effectiveness; (3) ICER between one and three times per capita GDP shows moderate cost-effectiveness; (4) ICER > 3 times per capita GDP suggests the program is not cost-effective due to high investment needs (29).

### 2.6 Statistics and ethics

The model construction and cost-effectiveness analysis were conducted using TreeAge Pro 2022. The analysis involved calculating total costs, total utilities, incremental costs, incremental utilities, and incremental cost-effectiveness ratios. This study was based on publicly available literature and simulated cohorts, did not involve human or animal experiments, and therefore did not require ethical approval.

## 3 Results

### 3.1 Cost-effectiveness analysis

**Figure 2:**
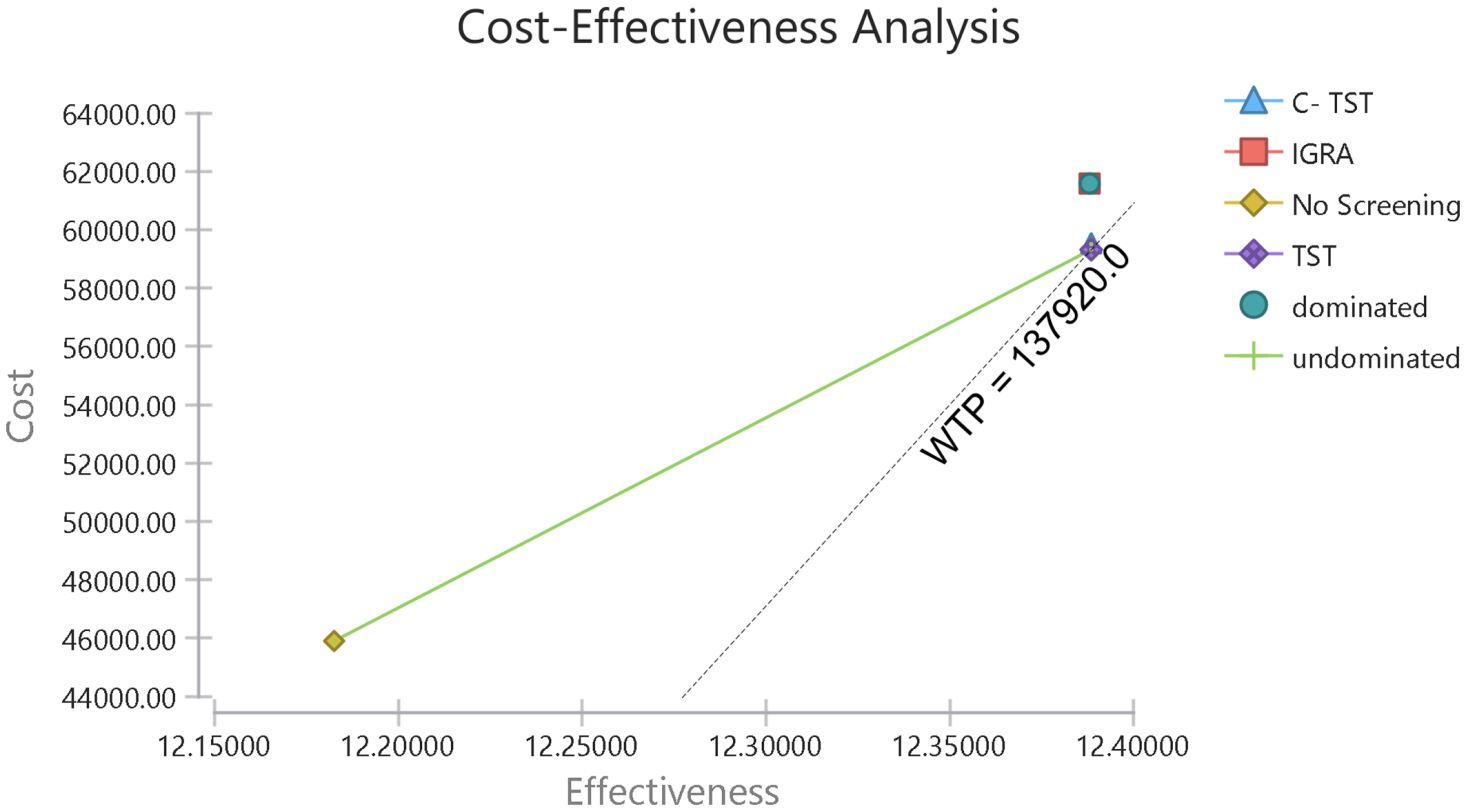
Cost-effectiveness analysis of three different screening strategies. IGRA, interferon-gamma release assay; C-TST, creation tuberculin skin test; TST, tuberculin skin test.

In the simulated cohort of 10,000 older diabetic individuals, the cost-effectiveness analysis indicated (Figure 2; Table 2) that the “No Screening” strategy had the lowest direct medical costs (45,910.8 yuan), while “IGRA” had the highest total cost (61,575.9 yuan) due to the reagent price exceeding 300 yuan. In terms of utility, C-TST had the highest nominal utility value (12.38847 QALYs), but its incremental utility compared to TST was less than 0.00003 QALY, lacking practical clinical significance. The incremental cost-effectiveness ratio (ICER) showed that, using “No Screening” as a reference, TST had the lowest ICER (65,083.6 yuan/QALY), significantly below the willingness-to-pay threshold (WTP = 137,920 yuan/QALY); C-TST’s ICER compared to TST was as high as 3,728,495.1 yuan/QALY, revealing its minimal incremental benefit. IGRA, due to its high cost, was the least economical option (ICER = 76,224.13 yuan/QALY).

**Table 2:** Cost-effectiveness analysis of three different screening strategies.

Dominance	Strategy	Cost (CNY)	Incremental cost (CNY)	Effectiveness (QALYs)	Incremental effectiveness (QALYs)	NMB (CNY)	ICER* (CNY/QALY)	ICER† (CNY/QALY)	ICER# (CNY/QALY)
Undominated	No screening	45,911		12.18253		16,34,304			
Undominated	TST	59,311	13,400	12.38842	0.20589	16,49,300	65,084		
Undominated	C- TST	59,513	203	12.38847	0.00005	16,49,105	37,28,495	37,28,495	66,052
abs. Dominated	IGRA	61,576	2,062	12.38805	−0.00043	16,46,984	−48,33,333	−60,84,885	76,224

abs.dominated, absolutely dominated; ICER*, incremental cost-effectiveness ratio compared to the previous preferred alternative; ICER†, Incremental cost-effectiveness ratio compared with TST; ICER#, incremental cost-effectiveness ratio compared to No screening; IGRA, interferon-gamma release assay; NMB, net monetary benefit; QALYs, quality-adjusted life years; TST, tuberculin skin test; C-TST, creation tuberculin skin test.

### 3.2 One-way sensitivity analysis

**Figure 3:**
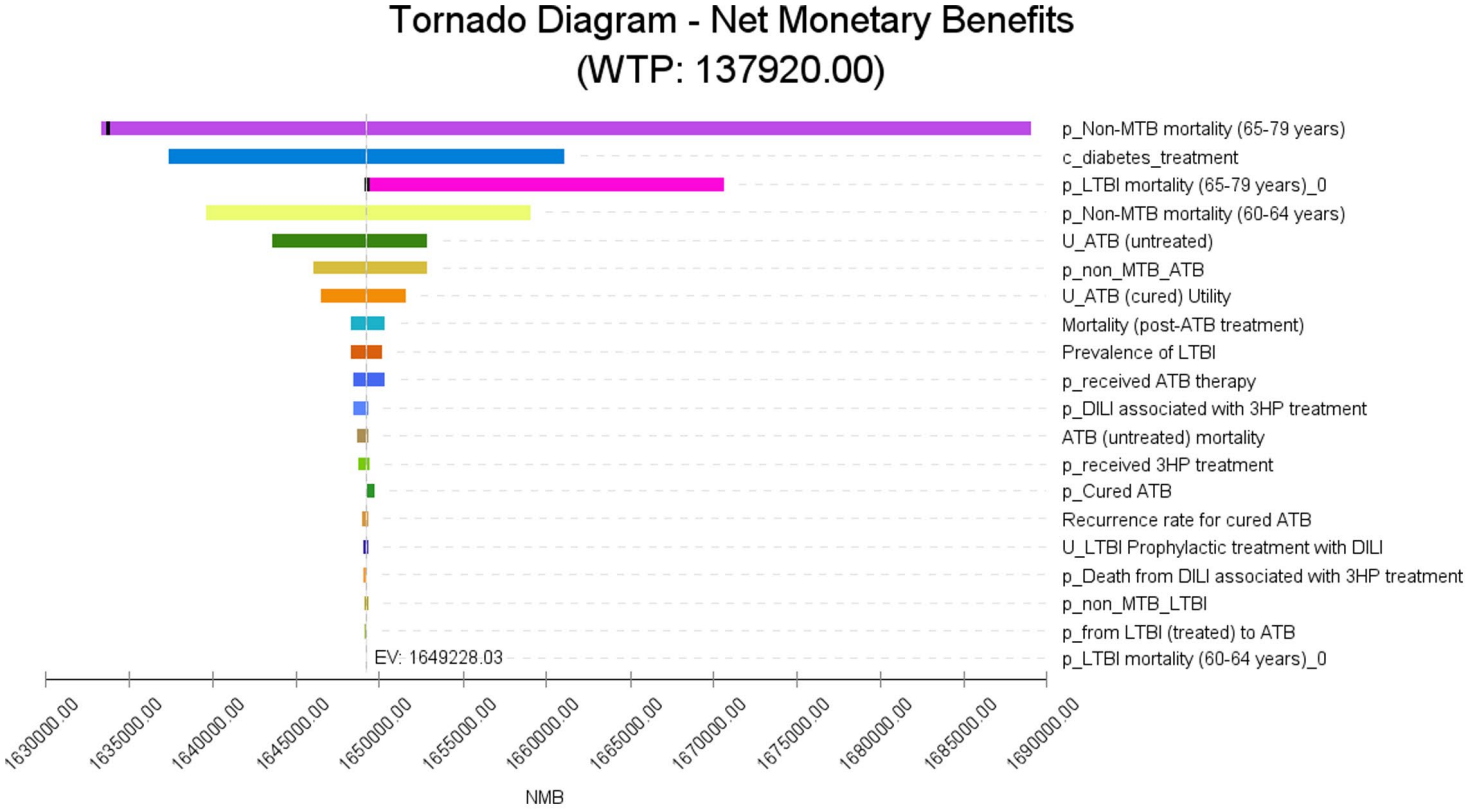
Tornado diagram of one-way sensitivity analysis (net monetary benefits). WTP, willingness-to-pay; NMB, net monetary benefits; EV, expected value; p_non_MTB mortality (65–79 years), non-MTB mortality for individuals aged 65 to 79 years; c_diabetes_treatment, annual medical costs for people with diabetes; p_LTBI_mortality (65–79 years), LTBI mortality for individuals aged 65 to 79 years; p_non_MTB mortality (60–64 years), non-MTB mortality for individuals aged 60 to 64 years; u_ATB (untreated), utility of untreated ATB; p_non_MTB_ATB, incidence of ATB in non_MTB cases; u_ATB (cured) utility, utility of cured ATB; Mortality (post-ATB) treatment, mortality for post-ATB treatment; Prevalence of LTBI, prevalence of individuals with LTBI; p_received ATB therapy, proportion of patients who received ATB therapy; p_DILI associated with 3HP treatment, proportion of DILI associated with 3HP treatment; ATB (untreated) mortality, mortality among untreated ATB cases; p_received 3HP_treatment, proportion of patients receiving 3HP treatment; p_cured ATB, proportion of cured ATB cases; Recurrence rate for cured ATB, recurrence rate among successfully treated ATB cases; u_LTBI prophylactic treatment with DILI, utility for treating LTBI with prophylactic treatment and developing DILI; p_death from DILI associated with 3HP treatment, proportion of mortality due to DILI associated with 3HP treatment; p_non_MTB_LTBI, incidence of latent tuberculosis infection in non_MTB individuals; p_from LTBI (treated) to ATB, probability of progression from treated LTBI to ATB; p_LTBI mortality (60–64 years), mortality of LTBI in individuals aged 60 to 64 years. ATB, active tuberculosis; Cured ATB, cured tuberculosis; LTBI, latent tuberculosis infection; Non-MTB, not infected with *Mycobacterium tuberculosis*; DILI, Drug-induced liver injury; 3HP treatment, isoniazid plus rifapentine administered once weekly for 12 weeks.

One-way sensitivity analysis indicated that key parameters affecting strategy economics included (Figure 3): non-tuberculous mortality rates for ages 65–79, LTBI mortality rates, annual medical costs for diabetes, non-tuberculous mortality rates for ages 60–64, and utility values for active tuberculosis. The core pattern was that when the non-tuberculous mortality rate reached 0.0359, all screening strategies became economically unviable. Increased mortality risk simultaneously raised total costs and lowered health utility, leading to diminished marginal benefits of screening. TST maintained a relatively favorable ICER only at low mortality rates (attributed to high treatment cost savings from preventing active tuberculosis), highlighting that comorbidity mortality rates are a core factor limiting the feasibility of screening (Supplementary Table S1).

### 3.3 Probabilistic sensitivity analysis and strategy selection

**Figure 4:**
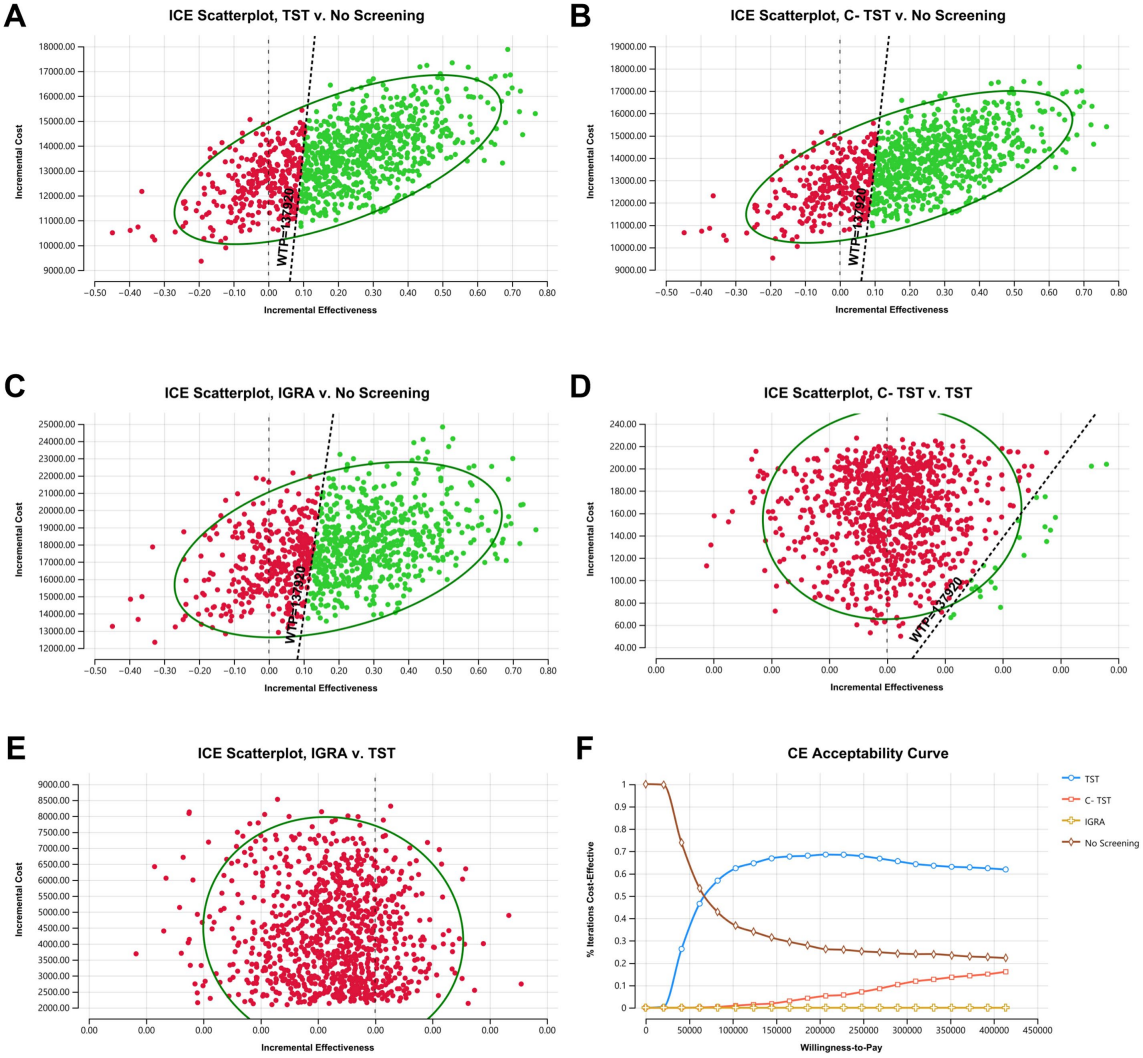
Probabilistic sensitivity analysis. **(A)** ICE scatterplot (TST vs. no screening); **(B)** ICE scatterplot (C-TST vs. no screening); **(C)** ICE scatterplot (IGRA vs. no screening); **(D)** ICE scatterplot (C-TST vs. TST); **(E)** ICE scatterplot (IGRA vs. TST); **(F)** cost-effectiveness acceptability curve. C-TST, creation tuberculin skin test; ICE, incremental cost-effectiveness; IGRA, interferon-gamma release assay; TST, tuberculin skin test.

After conducting 1,000 Monte Carlo simulations, it was found that the TST was cost-effective in 70.7% of the simulated scenarios, defined as having an ICER of less than one time the WTP (Figure 4A). In contrast, 14.8% of scenarios were deemed unacceptable due to high costs, and 14.5% demonstrated absolute disadvantages. The C-TST proved economical in 67.7% of simulations, with 18.1% revealing absolute disadvantages (Figure 4B). The IGRA had the lowest probability of being cost-effective at 64.1%, with 21.3% of simulations indicating excessively high costs, highlighting its price limitations (Figure 4C). Direct comparisons between these strategies revealed that C-TST was superior to TST in only 2.3% of the simulations, while 52.8% of scenarios were too costly. In contrast, IGRA showed absolute disadvantages in 83.8% of simulations, further highlighting its high-cost issues (Figures 4D,E). Acceptability curves (see Figure 4F) further confirmed that when the WTP exceeded 69,000 yuan per QALY, TST’s acceptance rate rapidly increased to 69.6% and continued to dominate at a WTP of 137,920 yuan, which aligns with the one-time per capita GDP threshold. In contrast, the maximum acceptance rate of C-TST was only 16.0% at a WTP of 413,760 yuan, while that of IGRA remained below 1%. At a WTP of 413,760 yuan per QALY, the acceptance probabilities for TST, C-TST, IGRA, and no screening were 61.8%, 16.0%, 0%, and 22.2%, respectively.

## 4 Discussion

This study evaluated the cost-effectiveness of three screening strategies for LTBI in the diabetic population aged 60 years or older in Fujian Province, China: traditional TST, new C-TST, and IGRA. The main findings are as follows: all three screening strategies demonstrated high economic feasibility, with ICERs below the WTP threshold. Traditional TST was the most cost-effective option, but had a high misdiagnosis rate. The new C-TST improved specificity at a slight economic cost, while IGRA had the highest specificity but lower cost-effectiveness due to its high price. Non-MTB mortality rates significantly affect the economic value of screening, as higher rates increase costs and decrease health utility.

The cost-effectiveness analysis results showed that the health utility values of patients undergoing the three screening programs are higher than those of patients without screening. This indicates that conducting LTBI screening for diabetes patients can effectively improve the quality of life of these patients. The results of this study align with previous research regarding the choice and necessity of screening strategies. The conclusions strongly support targeted screening in older diabetic patients (30, 31). This is consistent with previous studies indicating that diabetes significantly increases the risk of LTBI [adjusted odds ratio (aOR) = 1.18–1.21] (32, 33), and that the LTBI positivity rate is extremely high in specific populations (e.g., 23.6%–27.2% in Hispanic/Asian DM populations in the U.S.) (34). This study confirms the economic advantage of traditional TST under current resource allocation conditions in Fujian (lowest ICER), which conceptually aligns with practices in primary healthcare research in Malaysia that adjusted TST thresholds (8 mm) to optimize efficiency (31); however, this study further quantifies its cost-effectiveness in the older DM population.

However, there are significant differences and additions in the cost-effectiveness evaluation of new screening methods and handling of key parameters compared to previous studies. This study emphasizes that the high cost of IGRA is the primary reason for its limited utility, with its cost-effectiveness being lower than that of TST in grassroots settings. This contrasts with some views that IGRA is more suitable for resource-rich areas or immunocompromised populations due to its operational convenience (single blood draw, significantly reducing the burden of multiple visits for older individuals with mobility issues) (32, 33). This difference highlights the decisive role of resource environment and medical costs in such evaluations. Although global developments have produced C-Tb (Denmark), Dia skin test (Russia), and China’s EC as C-TST reagents—which have significantly lower costs than IGRA (approximately one-sixth of IGRA) and do not require laboratory support (35)—their safety and accuracy still need careful consideration (36). This study found that while the specificity of the new C-TST is superior to that of the traditional TST, its incremental utility is minimal, failing to demonstrate economic viability over the TST in the cost-effectiveness model.

This study is the first localized cost-effectiveness evaluation of LTBI screening strategies for the older diabetic population in China. The research relies on real grassroots cost data from the Fujian Sanming medical insurance payment reform pilot (local TST reagent prices are 30% lower than the national average), directly comparing three new and old strategies (TST, new C-TST, and IGRA), and clearly confirms that under similar resource allocation conditions (especially considering medical insurance cost control), TST has the optimal incremental cost-effectiveness (lowest ICER). This not only provides direct evidence-based support for prioritizing TST screening in older DM management in resource-limited areas (e.g., rural Fujian) (37, 38) but also echoes the WHO’s recommendation for context-specific strategies. Furthermore, the study identifies “non-tuberculous comorbidity mortality rates” as a core factor driving model uncertainty, strongly suggesting that future interventions should integrate LTBI screening into a broader framework of older comorbidity management (especially cardiovascular risk management).

Based on the above results, we recommend conducting screening programs in areas with limited grassroots resources, such as rural Fujian, by relying on existing general clinics, diabetes clinics, and grassroots medical and health institutions. Specifically, traditional TST screening can be incorporated into the basic management package for diabetes by adding an LTBI screening step in the routine follow-up process. This step should be completed by grassroots medical personnel, who have undergone standardized training at village health clinics or community health service centers for TST administration and result interpretation. This strategy actively responds to the national public health call for “tuberculosis-diabetes co-management” (39) and relies on a mature grassroots service structure, facilitating rapid implementation. In economically developed areas, consideration can be given to using IGRA in diabetes specialty or general outpatient clinics within secondary hospitals or higher-level hospitals that meet specific criteria, weighing the testing costs against the convenience of requiring only a single visit, which benefits patient time and resource utilization (40). For all regions planning to implement the new C-TST screening method, further control of reagent and operational costs should be implemented, along with strengthening adverse reaction monitoring and evaluation of the consistency of operational procedures.

To optimize the comprehensive management of chronic diseases such as diabetes, the “three doctors co-management” service model in Fujian can be utilized. In this model, health managers, general practitioners, and specialists work collaboratively to integrate LTBI screening with existing comorbidity intervention measures, such as annual health check-ups and cardiovascular risk assessments. By leveraging existing follow-up management pathways and information platforms, this approach enhances screening coverage efficiency and improves follow-up completion quality, thereby better controlling the risk of tuberculosis incidence and reducing overall mortality.

This study is subject to several limitations. First, the primary source of cost data is derived from Sanming City in Fujian Province, where medical insurance cost-control measures are notably stringent. Consequently, reagent costs, particularly for TST, may vary significantly—by a factor of two to three—in other regions. Additionally, substantial differences in the composition of healthcare resources across regions necessitate caution when extrapolating these findings. Second, this study does not distinguish between Type 1 Diabetes Mellitus (T1DM) and Type 2 Diabetes Mellitus (T2DM). Given that experimental studies indicate glycerol metabolism in T2DM, patients may facilitate the proliferation of tuberculosis bacteria (3). Future research should incorporate stratified analyses to address this differentiation. Third, the model employed in this study does not sufficiently account for the potential complex effects of other comorbid chronic diseases, particularly cardiovascular diseases, on the effectiveness of screening.

To deepen understanding of mechanisms and optimize policies, future research should consider the following aspects: First, conduct multi-regional cost-effectiveness analyses that incorporate regions with significant differences in reagent prices (e.g., non-medical insurance cost control pilot areas) to explore the universality and promotion thresholds of screening models under different cost structures. Second, conduct stratified studies on diabetes types, establishing subgroup models targeting the metabolic characteristics of T2DM patients to quantify their impact on LTBI risk and screening benefits. Third, explore comorbidity interactions by conducting in-depth studies on the interactions between common comorbidities, such as cardiovascular diseases, and LTBI screening and treatment. Fourth, perform dynamic policy simulation by developing a multidimensional decision model that incorporates regional costs, population stratification, and comorbidity risks to provide evidence-based support for differentiated medical insurance policies.

## 5 Conclusion

This study assesses the cost-effectiveness of LTBI screening strategies for older individuals with diabetes in China. Traditional TST was the most cost-effective option despite the risk of misdiagnosis, while new C-TST and IGRA were less effective due to their higher costs. It emphasizes TST’s suitability for resource-limited areas and suggests integrating LTBI screening with broader comorbidity management to reduce mortality. Recommendations include prioritizing TST, considering IGRA in wealthier regions, optimizing costs for C-TST, and linking screening with interventions for chronic diseases.

## Data Availability

The original contributions presented in the study are included in the article/Supplementary material, further inquiries can be directed to the corresponding author.

## Glossary

ATB: active tuberculosis
Cured ATB: cured active tuberculosis
CDC: Centers for Disease Control and Prevention
C-TST: new recombinant tuberculosis fusion protein skin test
DILI: drug-induced liver injury
DM: diabetes mellitus
EC: recombinant *Mycobacterium tuberculosis* fusion protein
GDP: gross domestic product
ICER: incremental cost-effectiveness ratio
IGRA: interferon-gamma release assay
LTBI: latent tuberculosis infection
MDR-TB: multi-drug resistant tuberculosis without extensive drug resistance
Non-MTB: non-infected with *Mycobacterium tuberculosis*
PPD: purified protein derivative
QALYs: quality-adjusted life years
QFT: QuantiFERON-TB Gold
TB: tuberculosis
TST: traditional tuberculin skin test
T-SPOT.TB: TB-specific enzyme-linked immunospot assay
WHO: World Health Organization
WTP: willingness to pay
2HRZE+4HR: two months of Isoniazid, Rifampin, Pyrazinamide, and Ethambutol, followed by four months of Isoniazid and Rifampin
3HP regimen: isoniazid plus rifapentine once a week for 12 weeks.
