# Supplementary material for: Cost-effectiveness analysis of tuberculosis screening in diabetic patients in China: a decision-analytic Markov model
Source: Front Public Health. 2026 Jan 5;13:1696952. doi: 10.3389/fpubh.2025.1696952 (PMC12813112; doi:10.3389/fpubh.2025.1696952)
Supplement: Supplementary file 1 [file Image_1.PDF]

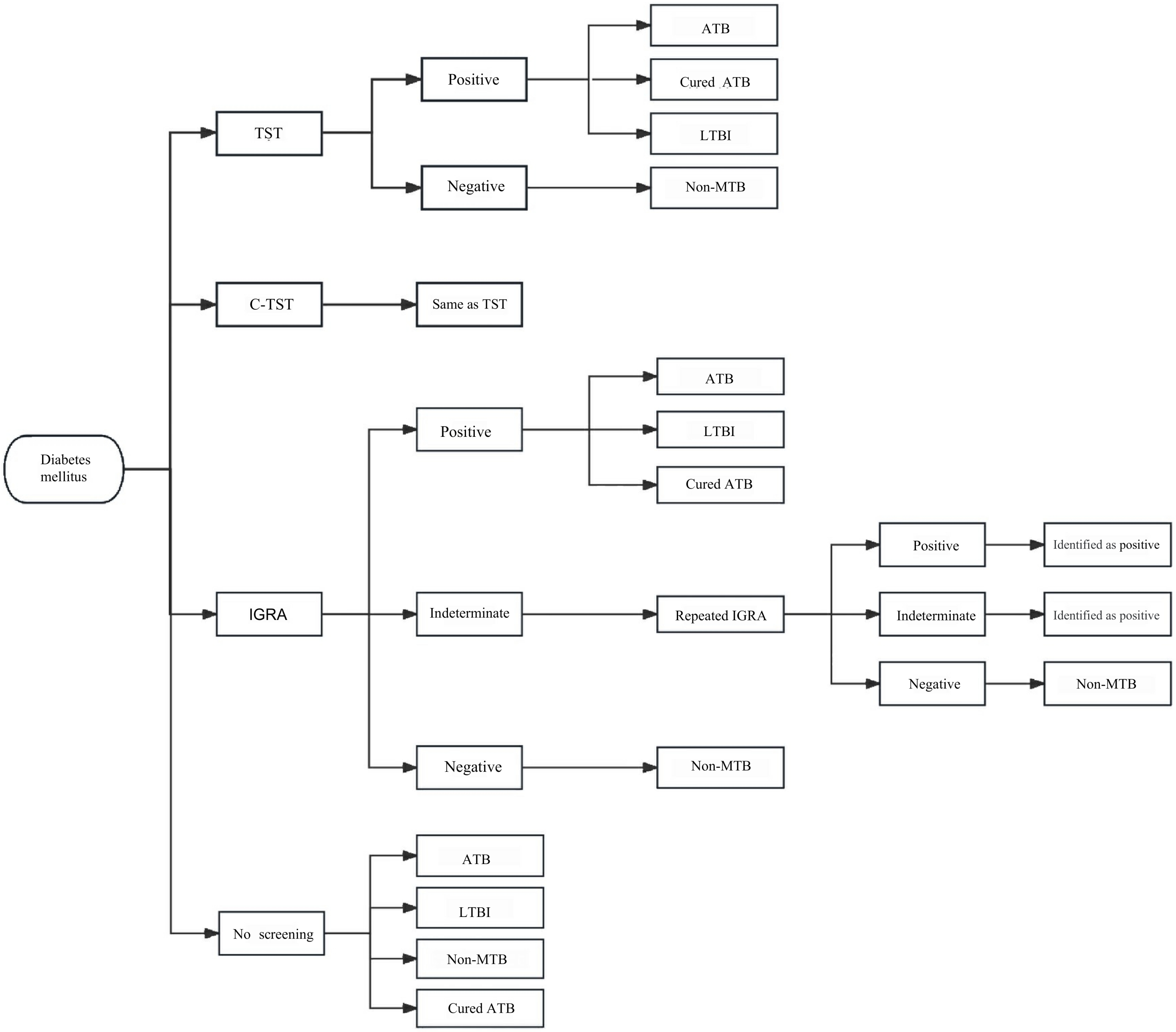

S.Figure 1 Clinical pathways for screening latent tuberculosis infection in diabetic patients.

ATB: active tuberculosis, Cured ATB: cured active tuberculosis, C-TST: new recombinant tuberculosis fusion protein skin test, IGRA: interferon-gamma release assay, LTBI: latent tuberculosis infection, Non-MTB: non-infected with Mycobacterium tuberculosis, TST: traditional tuberculin skin test.

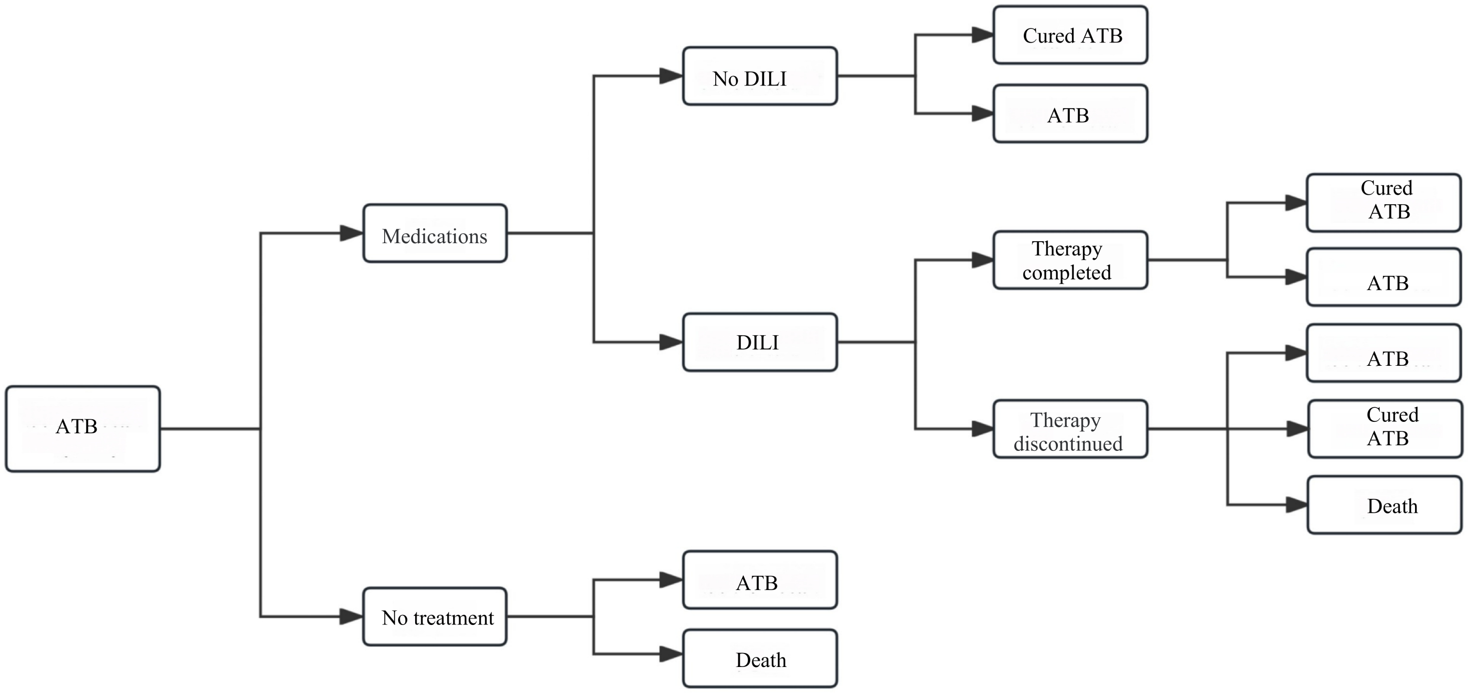

S.Figure 2 Treatment pathways for active tuberculosis.

ATB: active tuberculosis, Cured ATB: cured active tuberculosis, DILI, Drug-induced liver injury.

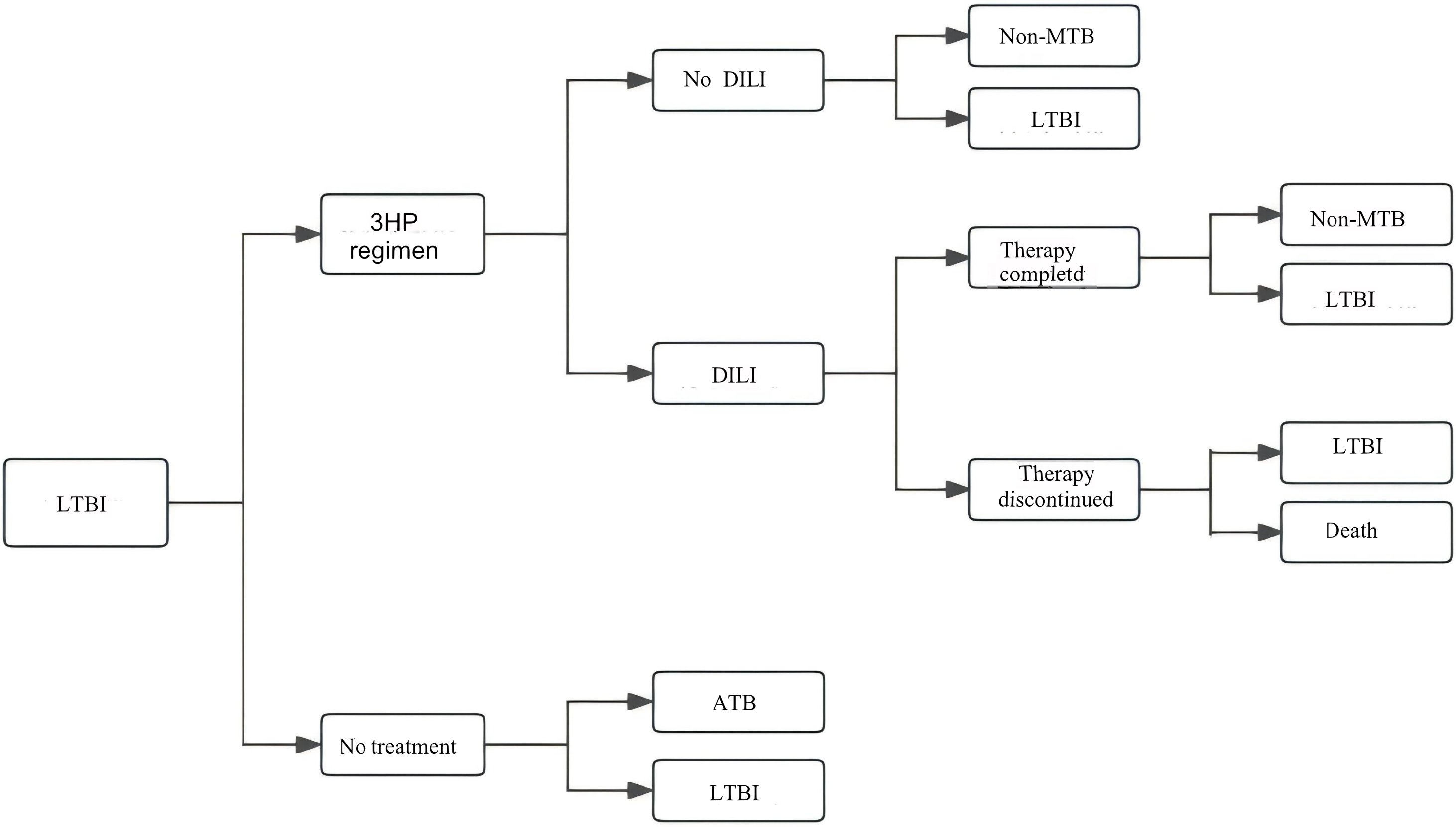

S.Figure 3 Preventive treatment pathways for latent tuberculosis infection.

ATB: active tuberculosis, DILI, Drug-induced liver injury, LTBI: latent tuberculosis infection, Non-MTB: non-infected with Mycobacterium tuberculosis, 3HP regimen, isoniazid plus rifapentine administered once weekly for 12 weeks.

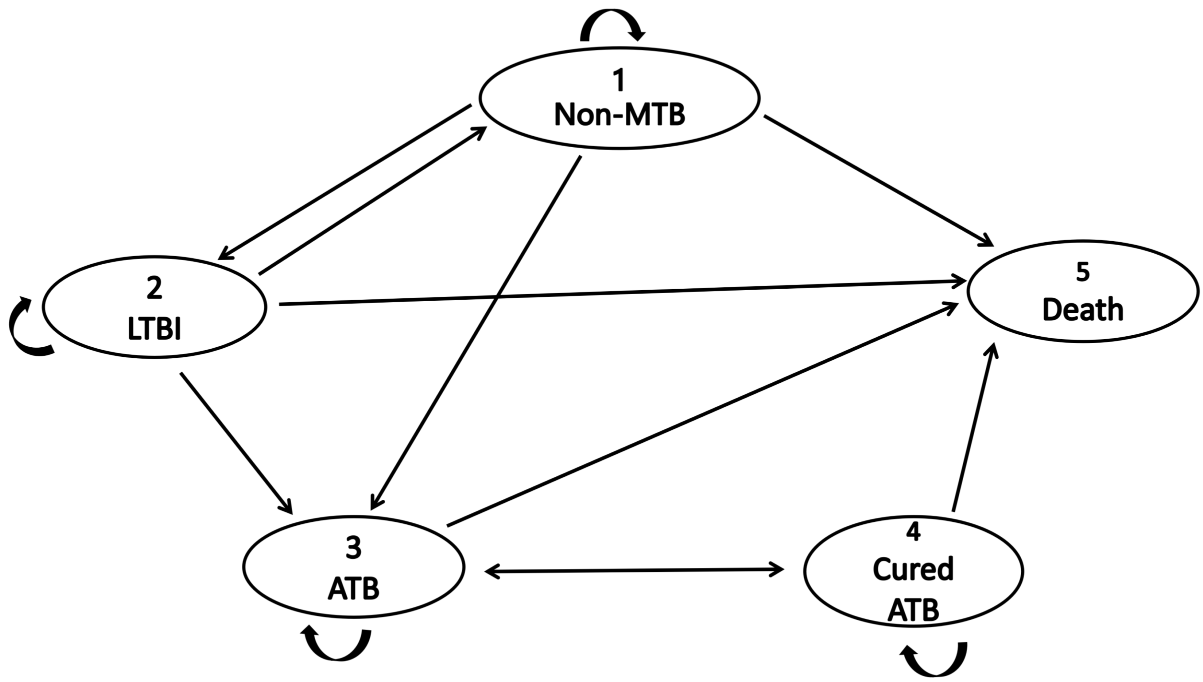

S.Figure 4 Natural history model of tuberculosis

ATB: active tuberculosis, Cured ATB: cured active tuberculosis, LTBI: latent tuberculosis infection, Non-MTB: non-infected with Mycobacterium tuberculosis.
